# Qualitative and quantitative analysis with a novel shear wave speed imaging for differential diagnosis of breast lesions

**DOI:** 10.1038/srep40964

**Published:** 2017-01-19

**Authors:** Yu-Ping Yang, Xiao-Hong Xu, Le-Hang Guo, Ya-Ping He, Dan Wang, Bo-Ji Liu, Chong-Ke Zhao, Bao-Ding Chen, Hui-Xiong Xu

**Affiliations:** 1 Department of Medical Ultrasound, Shanghai Tenth People’s Hospital, Ultrasound Research and Education Institute, Tongji University School of Medicine, Shanghai 200072, China; 2 Thyroid Institute, Tongji University School of Medicine, Shanghai 200072, China; 3 Department of Ultrasound, Guangdong Medical University Affiliated Hospital, Zhanjiang, 524001, China

## Abstract

To evaluate the diagnostic performance of a new two-dimensional shear wave speed (SWS) imaging (i.e. Toshiba shear wave elastography, T-SWE) in differential diagnosis of breast lesions. 225 pathologically confirmed breast lesions in 218 patients were subject to conventional ultrasound and T-SWE examinations. The mean, standard deviation and ratio of SWS values (m/s) and elastic modulus (KPa) on T-SWE were computed. Besides, the 2D elastic images were classified into four color patterns. The area under the receiver operating characteristic (AUROC) curve analysis was performed to evaluate the diagnostic performance of T-SWE in differentiation of breast lesions. Compared with other quantitative T-SWE parameters, mean value expressed in KPa had the highest AUROC value (AUROC = 0.943), with corresponding cut-off value of 36.1 KPa, sensitivity of 85.1%, specificity of 96.6%, accuracy of 94.2%, PPV of 87.0%, and NPV of 96.1%. The AUROC of qualitative color patterns in this study obtained the best performance (AUROC = 0.957), while the differences were not significant except for that of Eratio expressed in m/s (AUROC = 0.863) (*P* = 0.03). In summary, qualitative color patterns of T-SWE obtained the best performance in all parameters, while mean stiffness (36.05 KPa) provided the best diagnostic performance in the quantitative parameters.

Breast cancer is one of the most common cancers of women and its incidence rate is increasing in recent years^1^,^2^. Palpation plays a crucial role in detecting conspicuous breast lesions. Nevertheless, it has several limitations, such as lack of sensitivity and objectivity for the deep-situated or small lesions^3^. Ultrasound (US) can prominently visualize the target lesion through real-time scanning and give detailed information about the lesion. Breast cancers are generally stiffer than benign lesions. However, the stiffness information of lesions is not available with conventional US. In recent years, US elastography has been developed to evaluate the tissue stiffness. Alike conventional US, US elastography is non-invasive, free of radiation and easy to learn, therefore, it is widely used with an aim to improve the differentiation of breast lesions. Because of the anatomical advantages of breast, which is superficial and free of interfering factors such as carotid artery pulsation, the application of US elastography in breast has gained promising performance and effectively improved diagnostic confidence.

According to the recommended guideline for clinical use of elastography on breast, elastography techniques can be classified into the following main types^4^,^5^. The first is strain imaging which includes strain elastography (SE) and acoustic radiation force impulse (ARFI) imaging (i.e., virtual touch tissue imaging, VTI; Siemens Medical Solutions, Mountain View, CA, USA). SE provides qualitative or semi-quantitative evaluation and several studies reported that SE helps improve the diagnostic efficiency of conventional US in the differential diagnosis of breast lesions^6^,^7^,^8^. Nevertheless, the application of SE is limited for that it lacks quantitative information and is highly operator-dependent due to the reliance on external force^9^. Thereafter, ARFI imaging has been developed, which is based on ARFI displacement induced by ARFI excitation from the transducer and has less examiner-dependence and more reproducibility. However, ARFI imaging still cannot provide the quantitative evaluation of lesion stiffness. To overcome this limitation, shear wave imaging including point shear wave speed (SWS) measurement and SWS imaging has been introduced into clinical practice. Point SWS measurement such as virtual touch tissue quantification (VTQ; Siemens Medical Solutions, Mountain View, CA, USA) can measure the SWS of tissue and several studies showed that point SWS measurement is useful in differentiation between benign and malignant breast lesions^10^,^11^,^12^. Nonetheless, point SWS measurement is not able to provide two-dimensional (2D) stiffness distribution of target lesion. SWS imaging, which is also named as 2D shear wave elastography (SWE), not only provides 2D stiffness distribution but also expresses the tissue stiffness in kilopascals (KPa) or meters per second (m/s) based on different US machines.

Three main types of 2D SWS imaging techniques are applied in clinical practice, which are shear wave elastography (SWE) from Super Sonic Imagine (SSI; Aix en Provence, France), virtual touch tissue imaging & quantification (VTIQ; Siemens Medical Solutions, Mountain View, CA, USA) and Toshiba SWE (T-SWE; Toshiba Medical System, Tochigi, Japan). SWE from Super Sonic Imagine (SSI) can show the stiffness of tissues and the stiffness is expressed in KPa (range, 0–180 KPa) with promising performances^13^,^14^,^15^,^16^,^17^. The tissue stiffness on VTIQ is denoted by SWS distribution, which is expressed in m/s (range, 0.5–10 m/s) and has gained promising application in diagnosis of breast lesions^18^,^19^,^20^. In terms of the newly developed T-SWE, it can exhibit the stiffness distribution of lesions both in KPa (range, 0–180 KPa) and in m/s (range, 0.5–8.0 m/s). Until present, no study about T-SWE in breast lesion has been published. This study was aimed to evaluate the diagnostic performance of qualitative and quantitative analyses for T-SWE in the differentiation of breast lesions.

## Methods

### Patient enrollment

**Figure 1:**
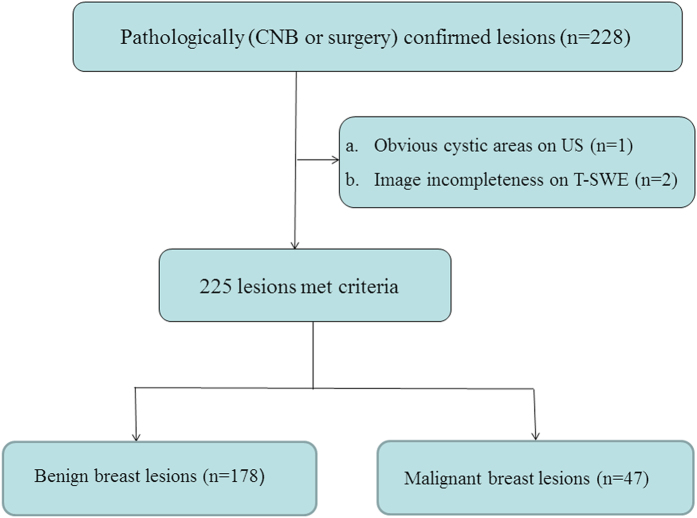
Flowchart for the selection of breast lesions.

This retrospective study was approved by the Ethics Committee of the Shanghai Tenth People’s Hospital of Tongji University School of Medicine and informed consent was waived for all the participating patients. From January 2016 to April 2016, a total of 250 consecutive patients with breast lesions underwent T-SWE examination in the university hospital. The inclusion criteria for the breast lesions were as follows: (a) breast lesions were palpable by clinicians or were visible on conventional US; (b) solid breast lesions or predominantly solid lesions (<25% cystic); (c) no previous treatment such as breast surgery, radiotherapy or chemotherapy was performed; (d) with pathological confirmation by ultrasound-guided core needle biopsy (CNB) or surgical excision. In this study, ultrasound-guided CNB or surgical excision was performed for breast lesions visible on ultrasound that had been assigned the Breast Imaging Reporting and Data System (BI-RADS)^21^ category 4 or 5 and category 3 at the request of patients or referring physicians. Ultimately, pathological confirmation was obtained in 228 lesions from 221 women. The exclusion criteria were as follows: (a) Obvious cystic areas on US (n = 1); (b) data incompleteness (n = 2). Finally, a total of 225 breast lesions in 218 women were included in this study (Fig. 1).

### Measurement protocol of T-SWE

**Figure 2:**
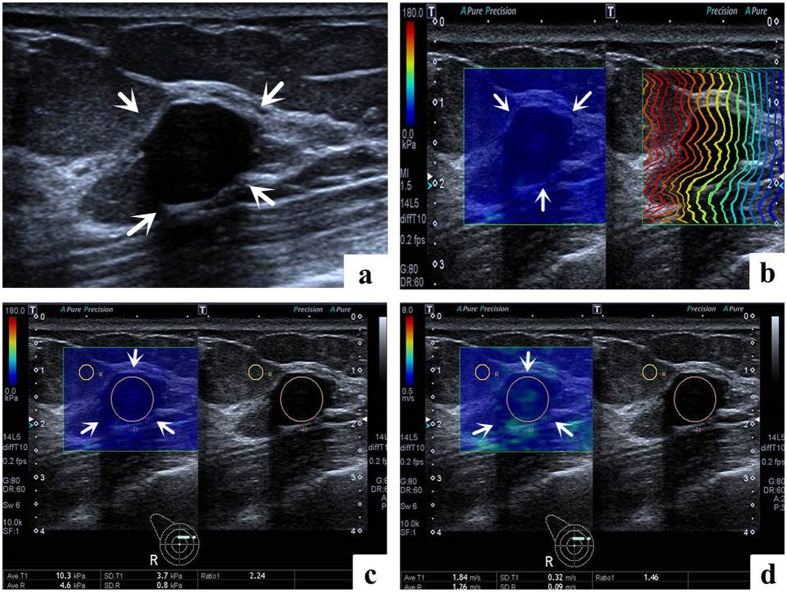
Images in a 50-year-old woman with fibroadenoma. (**a**) The lesion (arrows) is shown on B-mode ultrasound. (**b**) The lesion (arrows) shows regularly parallel contour lines on the shear wave propagation mode. (**c**) The Emean, E_SD_ and Eratio of the lesion (arrows) on elasticity mode are 10.3 kPa, 3.7 kPa and 2.24 respectively. (**d**) The Emean, E_SD_ and Eratio of the lesion (arrows) on shear wave speed mode are 1.84 m/s, 0.32 m/s and 1.46 respectively.

**Figure 3:**
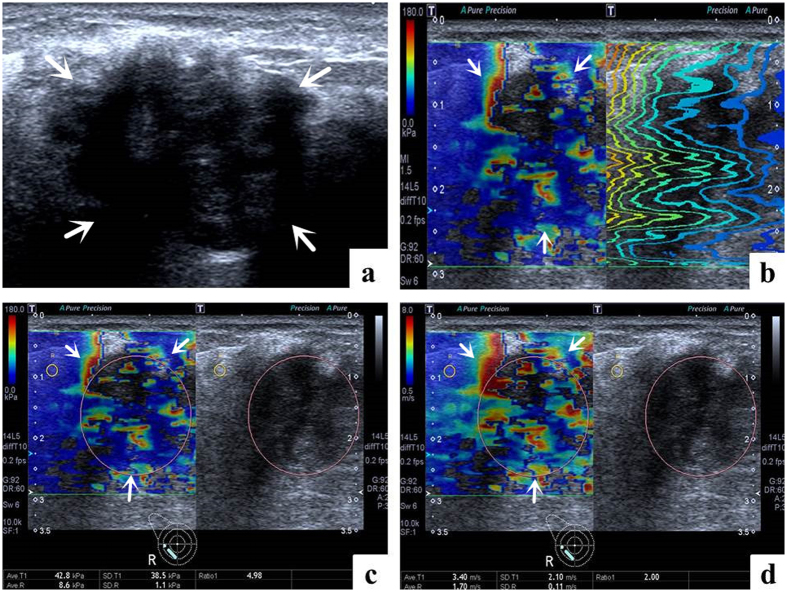
Images in a 62-year-old woman with invasive ductal carcinoma. (**a**) The lesion (arrows) is shown on B-mode ultrasound. (**b**) The lesion (arrows) shows irregularly distorted and chaotic contour lines on the shear wave propagation mode. (**c**) The Emean, E_SD_ and Eratio of the lesion (arrows) on elasticity mode are 42.8 kPa, 38.5 kPa and 4.98 respectively. (**d**) The Emean, E_SD_ and Eratio of the lesion (arrows) on shear wave speed mode are 3.40 m/s, 2.10 m/s and 2.00 respectively.

**Figure 4:**
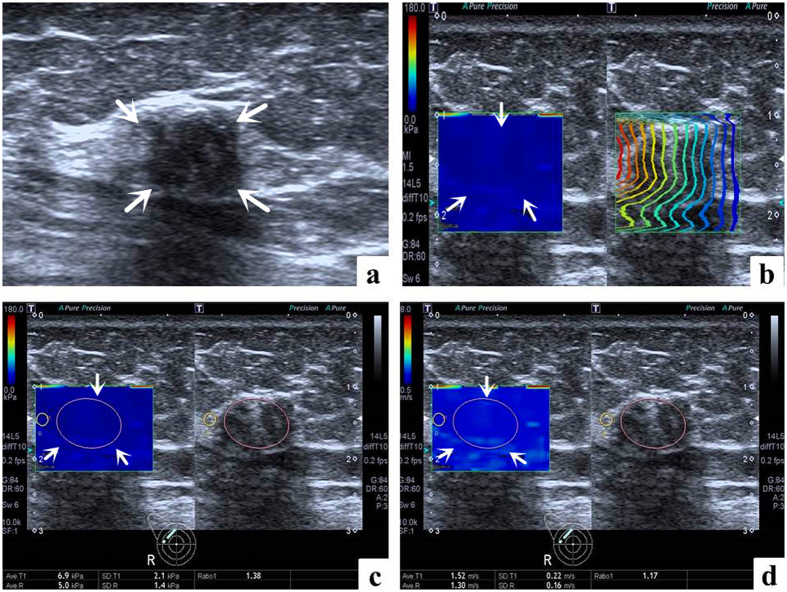
False negative case images in a 68-year-old woman with ductal carcinoma *in situ*. (**a**) The lesion (arrows) is shown on B-mode ultrasound. (**b**) The lesion (arrows) shows regularly parallel contour lines on the shear wave propagation mode. (**c**) The Emean, E_SD_ and Eratio of the lesion (arrows) on elasticity mode are 6.9 kPa, 2.1 kPa and 1.38 respectively. (**d**) The Emean, E_SD_ and Eratio of the lesion (arrows) on shear wave speed mode are 1.52 m/s, 0.22 m/s and 1.17 respectively.

**Figure 5:**
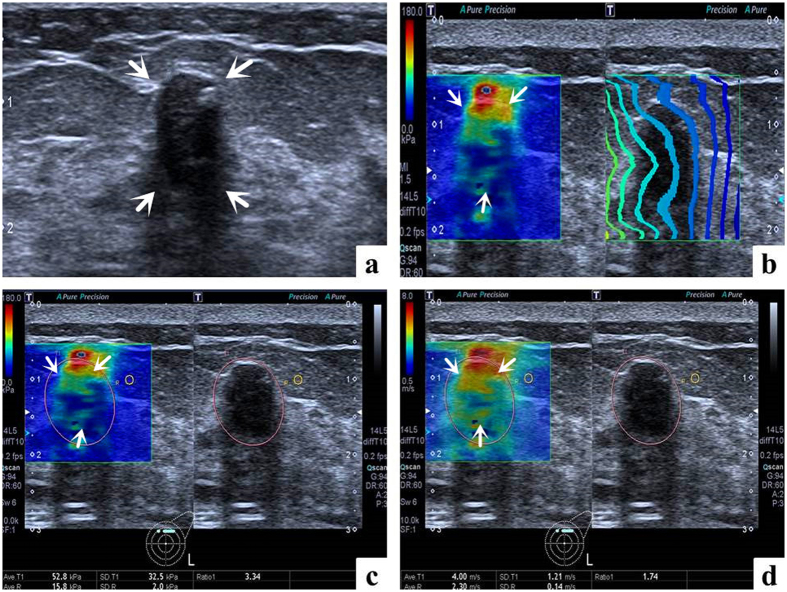
False positive case images in a 62-year-old woman with fibroadenomatous hyperplasia. (**a**) The lesion (arrows) is shown on B-mode ultrasound. (**b**) The lesion (arrows) shows irregularly distorted and chaotic contour lines on the shear wave propagation mode. (**c**) The Emean, E_SD_ and Eratio of the lesion (arrows) on elasticity mode are 52.8 kPa, 32.5 kPa and 3.34 respectively. (**d**) The Emean, E_SD_ and Eratio of the lesion (arrows) on shear wave speed mode are 4.00 m/s, 1.21 m/s and 1.74 respectively.

Conventional US and T-SWE examinations were carried out with the same Aplio500 US machine (Toshiba Medical Systems Corporation, Tochigi, Japan) by one of two board-certified radiologists with more than 2 years of experience in breast US and elastography. Firstly, using a 14L5 liner array transducer (frequency range, 5–14 MHz), all patients were scanned in a supine position to obtain the transverse and longitudinal US images for the target breast lesion. Subsequently, T-SWE examinations were conducted with the same transducer at the longest length of breast lesions. The transducer was applied with an adequate amount of contact gel and kept vertical to the target lesion with pressure as slight as possible. The shear wave images were obtained with the patients holding breath for a few seconds under “One Shot Scan” mode (Figs 2, 3, 4 and 5). The interval between two performances is 5 seconds. Display modes can be switched among three options with freedom after image frozen, which include speed mode, elasticity mode, and propagation mode.

In the speed mode, it displays SWS with an SW-V map in m/s (range, 0.5–8.0 m/s), while in the elasticity mode; it displays modulus of elasticity with an SW-E map in KPa (range, 0–180 KPa). Both the SW-V map and SW-E map were obtained to display SWS distribution in semi-transparent 2D-colour imaging, which were overlaid on the B-mode image. The elasticity modulus or SWS is displayed by the gradual colors of these two maps with increasing stiffness revealed in ascending order of blue, green, yellow and red. Regions which are not color-coded on the elasticity images reflect that no shear wave is detected.

Regarding to the propagation mode, it displays arrival time contour with the shape of contour lines, through which operators preliminarily confirm whether shear waves propagate as expected and verify the reliability of the obtained data. When the contour lines are nearly straight and regularly parallel to each other, the reliability of data is high. On the contrary, when the contour lines are irregularly distorted and chaotic, the reliability of data is low. When the latter is encountered, it is necessary to perform another elastography to gain reliable result. The width of the intervals between the contour lines increases in stiff tissue while decreases in soft tissue. The propagation mode also provides guidance to evaluate whether region of interest (ROI) placement is accurate or not on speed mode or elasticity mode. In general, the ROI of the target lesion should be placed on areas with parallel contour lines.

The SWS values were measured by placing an ROI which can be adjusted according to the shape of target lesion in both SW-V map and SW-E map. Regarding to the placement of ROI, those influencing factors such as calcification, cystic portions, surrounding breast or fatty tissue which might lead to confusing results of elastic measurement should be avoided as far as possible. One ROI was placed in the target lesion and the other in the breast fatty tissue. Then the system automatically calculated the mean elasticity (Emean), standard deviation of the elasticity (E_SD_) and the ratio elasticity (Eratio) between the mean value in the lesion and in the fatty tissue in KPa and m/s.

**Figure 6:**
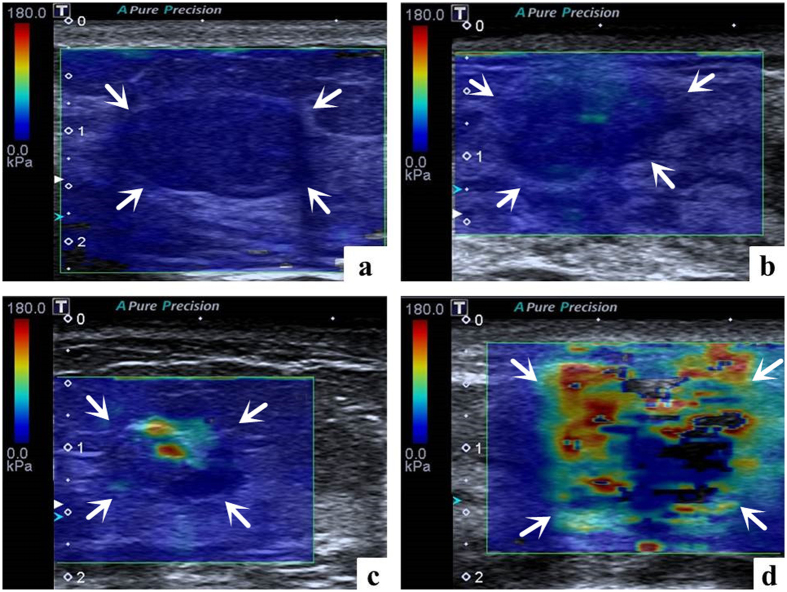
Solid breast lesions with four T-SWE color patterns on elasticity mode. (**a**) Pattern 1, the lesion is shown homogeneously light blue, classified as ‘negative results’. The diagnosis after surgical excision is fibroadenomatous hyperplasia. (**b**) Pattern 2, the lesion is shown heterogeneously, predominantly blue with spot green, classified as ‘negative results’. The diagnosis after surgical excision is adenosis. (**c**) Pattern 3, the lesion is shown heterogeneously, with patchy green, yellow and red, classified as ‘positive results’. The diagnosis after surgical excision is intraductal carcinoma. (**d**) Pattern 4, the lesion is shown extremely heterogeneously, multicolored with red, orange, green, blue and irregular areas without colors, which can be named ‘multicolored sign’, classified as ‘positive results’. The diagnosis after surgical excision is invasive ductal carcinoma.

Additionally, the 2D elastic images on elasticity mode were reviewed in consensus by 2 other radiologists with more than 3 years of experience in elastography. Inconsistent results were solved by the superior radiologist. The patient clinical information, other elastic results, and pathological results were hidden. Regarding the qualitative T-SWE features, as we mentioned above, in SW-E map, the gradual colors display the elasticity modulus from high (red) to low (blue) color. Thereafter, the 2D elastic images on elasticity mode of the breast lesions were classified into the following four color patterns: pattern 1, the lesion was shown homogeneously light or dark blue, classified as ‘negative results’; pattern 2, the lesion was shown heterogeneously, predominantly blue with spot-like green or orange, classified as ‘negative results’; pattern 3, the lesion was shown heterogeneously, with patchy green, yellow or red. classified as ‘positive results’; pattern 4, the lesion was shown extremely heterogeneously, multicolored with red, orange, green, blue and irregular areas without colors, which can be named ‘multicolored sign’, classified as ‘positive results’ (Fig. 6).

### Inter-observer and intra-observer agreement for qualitative T-SWE color patterns

To assess inter-observer agreement, another 30 consecutive patients with breast lesions were enrolled, and 2D T-SWE color patterns of the same lesion were independently evaluated by 2 operators with same experience on the same day. Regarding to the intra-observer agreement, repeated 2D T-SWE imaging of the same lesion was evaluated by the same operator on 2 different days.

### Inter-operator and intra-operator consistency of quantitative T-SWE in Emean

As regard to inter-operator consistency of quantitative T-SWE in Emean, another consecutive thirty breast lesions were enrolled in the evaluation conducted by another two independent operators who had similar experience on SWE. To evaluate the intra-operator consistency, it was tested by the same operator and the same performance was repeated with one day interval. All performances were conducted with the same method mentioned above and the cases were excluded in the final diagnostic efficiency analysis.

### Statistical analysis

All statistical analyses were performed with the SPSS 17.0 software (SPSS, Chicago, IL). The total lesions were divided into three subgroups according to lesion size: group 1: maximum diameter, ≤10 mm; group 2: maximum diameter, 10–20 mm; group 3: maximum diameter, >20 mm. Means and standard deviations were expressed for continuous data while counts and percentages for categorical data. The Emean, E_SD_, and Eratio in KPa and m/s were collected, and their means between benign and malignant lesions were compared using independent-samples *t* test. The chi-squared test was used in comparing pattern classification between benign and malignant lesions. The SWS values referring to pathological results were collected to construct receiver operating characteristic curve (ROC) in order to acquire area under ROC (AUROC), sensitivity, specificity, accuracy, positive predictive value (PPV) and negative predictive value (NPV). The best cut-off value of each parameter was obtained by using the maximum Youden index (sensitivity +specificity −1). Comparisons of AUROC were conducted to evaluate the diagnostic performance of each elasticity parameters. Comparisons of AUROC were estimated by univariate *Z* score test. Differences in sensitivity, specificity, accuracy, PPV and NPV were compared using the McNemar test or chi-square test. Intra-observer and inter-observer agreement for qualitative 2D T-SWE were evaluated by weighted κ statistics. Agreement was graded as poor (κ < 0.20), moderate (κ = 0.20–0.40), fair (κ = 0.40–0.60), good (κ = 0.60–0.80), and very good (κ = 0.80–1.00). Additionally, the inter-operator and intra-operator consistency of quantitative T-SWE were evaluated with the intra-class correlation coefficient. In this study, differences were considered to be statistically significant at a two-tailed *p* value < 0.05.

## Results

### Basic Characteristics

Ultimately, 218 women (mean age, 45.3 years ± 14.6; range, 15–85 years) with 225 breast lesions (mean size, 15.7 mm ± 9.0; range, 5–62 mm) were included. Of the 225 breast lesions, 178 (79.1%) were benign and 47 (20.9%) were malignant. Forty one (18.2%) lesions were confirmed by US-guided CNB and 184 (81.8%) were confirmed by surgery. The pathological results of the 225 breast lesions were shown in Table 1.

### Diagnostic performances of T-SWE

All parameters were significantly higher in malignant lesions compared with those in benign lesions in both T-SWE with KPa and T-SWE with m/s (all *P* < 0.001, Table 2).

In terms of differentiation of breast lesions, AUROC curves were constructed to determine the optimal cut-off values and then to obtain the sensitivity, specificity, accuracy, PPV and NPV for the T-SWE parameters (Table 3). All the AUC comparisons of quantitative parameters did not show significant differences between T-SWE with KPa and T-SWE with m/s. Compared with other quantitative T-SWE parameters, mean value expressed in KPa had the highest AUROC of 0.943 and the optimal cut-off value was 36.05 KPa, with corresponding sensitivity of 85.1%, specificity of 96.6%, accuracy of 94.2%, PPV of 87.0%, and NPV of 96.1%. The AUROC of mean value expressed in KPa in the three subgroups did not show significant difference (Table 4).

In addition, the AUROC of qualitative color patterns obtained the highest performance compared with Eratio in speed mode (*P* = 0.03 < 0.05). Of the 225 breast lesions, 117 (52.0%) were assigned to pattern 1 on qualitative analysis, 61 (27.1%) to pattern 2, 12 (5.3%) to pattern 3 and 35 (15.6%) to pattern 4. The malignancy rate of each pattern was 1.7% (2 out of 117), 4.9% (3 out of 61), 58.3% (7 out of 12), and 100.0% (35 out of 35), respectively. Significant difference was present in the comparison of color patterns between benign and malignant lesions, (*P* < 0.001) (Table 1). Compared with Emean expressed in KPa, color patterns improved the specificity and sensitivity, which could avoid one unnecessary biopsy for the benign cases and avoid missing two carcinomas for the malignant cases.

### Inter-observer and intra-observer agreement for qualitative T-SWE color patterns

The κ values were 0.782 (95% CI: 0.555–0.948) for inter-observer agreement and 0.832 (95% CI: 0.639–1.000) for intra-observer agreement (both *P* < 0.001).

### Inter-operator and intra-operator consistency of quantitative T-SWE in Emean

Emean expressed in KPa was used to evaluate the inter-operator and intra-operator consistency of T-SWE. The correlation coefficient was 0.894 (95% CI: 0.775–0.995) for inter-operator consistency while 0.948 (95% CI: 0.884–0.982) for intra-operator consistency.

## Discussion

Shear wave imaging has better performance compared with SE in that it provides quantitative stiffness information of the target lesion. T-SWE, as a new 2D-SWE and SWS imaging technique, emerges to be a useful modality to offer both quantitative and qualitative stiffness information for the differential diagnosis of breast lesions.

It is well known that stiffness of the tissue can be estimated and expressed in KPa by a physical quantity called modulus of elasticity (E) or Young’s modulus, which can be simply defined as the ratio between the applied stress and the induced strain according to the Hooke law. As the stress is hard to calculate, the accurate data of E is unknown. However, the shear wave propagation speed can be calculated by displacement of localized tissue under short-duration acoustic radiation force, thus the US elastography system can measure the elasticity of the tissue through the following formula: E = 3ρc^2^, in which ρ is the density of tissue expressed in kg/m^3^, c is the shear wave propagation speed expressed in m/s. Thus, speed is proportionally related to E. On the basis of this principle, T-SWE can not only offer qualitative 2D shear wave (SW) color maps but also quantitative elastic information expressed in both KPa and m/s.

As mentioned above, there are four elastic parameters provided by T-SWE, which are quantitative Emean, E_SD_, Eratio and qualitative SW color patterns. Regarding to quantitative parameters, Emean measures the general stiffness of the target tissue, while Eratio represents the relative stiffness of the lesion with a coherent elastic value. Malignant tissue is more heterogeneous than benign one, and E_SD_ shows the internal heterogeneity of the lesion^17^, therefore higher E_SD_ is linked to a higher risk of malignancy. The mean of each quantitative parameter showed a significant difference between malignant and benign breast lesions in T-SWE with KPa and T-SWE with m/s respectively (all *P* < 0.001), which was concordant with previous studies using other SWS imaging techniques^20^,^22^,^23^. In terms of qualitative parameter, color pattern shows the whole stiffness distribution of the lesion with gradual colors.

Among the three quantitative parameters of T-SWE, Emean showed the highest AUC in this study with cut-off value of 36.05 KPa. Several studies had different optimal cut-off values using Emean with different SWS imaging techniques. Evans *et al*.^22^,^24^ proposed using Emean of 50 KPa, Lee *et al*.^25^ proposed using Emean of 68.40 KPa, while Chang *et al*.^15^ proposed using Emean at a higher level of 80.17 KPa. Given that there is no report of T-SWE for reference up to now, one of the reasons leading to the difference in cutoff value might be that the percentage of malignancy in this study was lower (i.e. 20.9%). Additionally, the signal loss like “zero signal intensity” in the elasticity mode or the speed mode will diminish the E-mean values, making the cut-off value of E-mean relatively lower. Of course, difference might also be caused by different machines since the imaging principles are different.

In addition to Emean, E_SD_ showed excellent performance to display the stiffness distribution of lesion with a cut-off value of 17.90 KPa, which was a little higher to 12.1 KPa in a study by Gweon *et al*.^17^, indicating that the performance of E_SD_ was in concordance with the previous study. E_SD_ can show the internal heterogeneity of the lesion and E_SD_ expressed in KPa obtained the highest sensitivity, specificity, accuracy, PPV and NPV, which makes it a useful supplement to the above mentioned elasticity parameters.

As regard to the relative stiffness value, the best cutoff value of Eratio was 3.67, which was a little lower than 4.39 reported in the previous study^25^. Zhou *et al*.^26^ reported that fatty tissue was more stable than breast glandular tissue because fatty tissue is influenced little by the factors such as menstrual cycle, sex hormones and duration period. Nevertheless, in this study, the AUC of Eratio was relatively low compared with Emean; the reason might be that the individual difference still remained due to the different thickness of breast fatty tissue.

All the diagnostic performances of quantitative parameters of T-SWE were good, of which the AUCs ranged from 0.863 to 0.943 and did not show significant differences between T-SWE in KPa and T-SWE in m/s. These results were quite reasonable due to the direct link between Young modulus and the SWS. Moreover, all these T-SWE parameters were derivate from the same breast lesion. As we know, hard lesions were associated with malignancy, which showed high Emean and Eratio values and tended to be histologically heterogeneous which was represented by high E_SD_. Significant difference of mean values between malignant and benign lesions for quantitative parameters might support these results as well. In this study, inter- and intra- operator consistencies of T-SWE were well, implying the initial experience of T-SWE in differential diagnosis of breast was promising with the high reproducibility.

Qualitative SW color patterns showed the best performance with the optimal cut-off pattern set at 2–3. Thirty-two out of 35 invasive ductal carcinomas (IDCs) were classified into pattern 4 in this study, indicating malignant lesions were more heterogeneous, which could be verified by the good performance of E_SD_ values. The 2D map on elasticity pattern of IDCs seemed to be more prone to show the multicolored sign instead of almost black coded images resulted from the acoustic attenuation. And a larger sample size is needed to study whether or not the malignancy is more likely to show multicolored sign on elasticity pattern. Besides, inter-observer and intra-observer agreement for T-SWE color patterns were good; indicating that SW color patterns can qualitatively provide intuitive and convenient stiffness information for the investigators.

In this study, one false positive case was resulted from special location of the target lesion, which was close to the axilla. The final pathological result was fibroadenoma, but the Emean values were relatively high. In this study the size of the breast lesion did not influence the diagnostic efficiency of elastography using mean value expressed in KPa, which was concordant with the study by Sadigh G *et al*.^27^. Whereas, Yoon *et al*.^28^ showed that large lesion size, depth of breast and breast thickness influenced false-positive SWE features through multivariate analysis. Due to the confined and uneven location like axilla with little supernumerary breast tissue, the upper arm of patient needed to be stretched and then the performance of elastography might be influenced because of the variation of precompression^29^.

In addition, there were two kinds of situations leading to false negative results. On the one side, the E-mean value decreased because of the signal loss like “zero signal intensity”. On the other side, smaller malignant masses such as early stage breast cancer, carcinoma with internal necrosis, ductal carcinoma *in situ* (DCIS) and mucinous carcinoma might be soft. Due to the fact that SWE provides the stiffness information of lesions, therefore benign elastography features among those carcinomas might lead to false-negative SWE patterns. In this study, two IDCs sized 10mm and 11mm were misdiagnosed. Vinnicombe *et al*.^30^ reported soft invasive cancers were frequently small, which would lead to false-negative SWE results. Besides, one DCIS was displayed soft. Several studies^31^,^32^ proved that compared with IDC, SWE values in DCIS were relatively lower. It might be assumed that DCIS consisted of relatively softer tissues. There were only two DCIS cases in this study and further studies with a larger population were anticipated to verify this hypothesis.

Even though the diagnostic performances of different T-SWE parameters were good, there were several limitations in this study: Firstly, the sample size of this retrospective study was small, especially for malignant lesions. Secondly, 34 out of the 178 (19.1%) benign breast lesions were confirmed by US guided CNB. Although US guided CNB has been proven to be an accurate and safe alternative to surgical biopsy for diagnosis, the percentage of missed cancer of US guided CNB benign result was reported to be about 0.8%^33^, which might have little effect on the results of our study. Last but not least, due to this study was retrospective; it was really a shortcoming for no e-max value evaluation. E-mean value in this study had some shortcomings and just gave partial details about elastic information of breast lesions. Until present, no study about T-SWE has been published. This study was the first T-SWE application in breast lesions in one single institution with good diagnostic performance. Therefore, further prospective study with multicenter collaborations and long-term follow-up is needed to offer more confirmatory evidence to verify our results in the future. Moreover, although no studies about T-SWE for other organs such as thyroid, liver and prostate have been reported, it is anticipated that its use in these areas will gain attention in the recent future.

In conclusion, T-SWE is a useful diagnostic tool for differentiation of breast lesions. Both qualitative and quantitative analyses have good diagnostic performance and the reproducibility of T-SWE is also favorable.

## Additional Information

**How to cite this article:** Yang, Y.-P. *et al*. Qualitative and quantitative analysis with a novel shear wave speed imaging for differential diagnosis of breast lesions. *Sci. Rep.* **7**, 40964; doi: 10.1038/srep40964 (2017).

**Publisher's note:** Springer Nature remains neutral with regard to jurisdictional claims in published maps and institutional affiliations.

## Figures and Tables

**Table 1:** Distribution of different pathology results of 225 breast lesions by qualitative color patterns on T-SWE.

Pathology	NO.	T-SWE Color patterns			
		Pattern 1	Pattern2	Pattern3	Pattern 4
Malignant*	47	2	3	7	35
Invasive ductal carcinoma	36	1	1	2	32
Intraductal carcinoma	4	0	0	2	2
Invasive lobular carcinoma	2	0	0	1	1
Ductal carcinoma *in situ*	2	1	0	1	0
Entity papillary carcinoma	2	0	2	0	0
Apocrine carcinoma	1	0	0	1	0
Benign	178	115	58	5	0
Fibroadenomatous hyperplasia	89	58	29	2	0
Fibroadenoma	33	18	12	3	0
Adenosis	42	32	10	0	0
Inflammation change	5	2	3	0	0
Intraductal papilloma	3	2	1	0	0
Benign phyllodes tumor	1	0	1	0	0
Tubular adenoma	1	0	1	0	0
Stromal fibrosis	1	1	0	0	0
Ductal hyperplasia	2	1	1	0	0
Venous hemangioma	1	1	0	0	0

^*^In comparison with benign lesions, significant difference is present (*P* < 0.001).

**Table 2:** Comparison of different quantitative parameters between malignant and benign breast lesions for T-SWE.

Variables		Overall (n = 225)		P*	≤10 mm (n = 67)		P*	10–20 mm (n = 108)		P*	>20 mm (n = 50)		P*
		Benign (n = 178)	Malignant (n = 47)		Benign (n = 60)	Malignant (n = 7)		Benign (n = 92)	Malignant (n = 16)		Benign (n = 26)	Malignant (n = 24)	
Emean	kPa	15.90 ± 10.07(5.70–89.40)	64.66 ± 28.01(8.60–130.80)	<0.001	13.31 ± 5.99(5.70–46.10)	47.13 ± 30.07(12.90–101.80)	<0.001	16.63 ± 10.83(6.10–89.40)	65.53 ± 26.30(8.60–96.80)	<0.001	19.28 ± 13.23(6.40–59.30)	69.19 ± 27.69(19.40–130.80)	<0.001
	m/s	2.19 ± 0.59(1.39–5.82)	4.48 ± 1.32(1.70–7.65)	<0.001	2.04 ± 0.39(1.39–3.70)	3.85 ± 1.55(2.08–6.70)	<0.001	2.24 ± 0.64(1.40–5.82)	4.44 ± 1.20(1.70–5.98)	<0.001	2.36 ± 0.74(1.42–4.24)	4.68 ± 1.32(2.01–7.65)	<0.001
E_SD_	kPa	5.72 ± 5.02(0.60–35.10)	39.91 ± 13.84(1.10–54.80)	<0.001	4.34 ± 4.49(0.60–28.10)	25.09 ± 19.35(1.10–54.80)	<0.001	5.82 ± 4.05(0.60–30.10)	38.39 ± 12.51(1.20–53.20)	<0.001	8.58 ± 7.61(1.90–35.10)	45.25 ± 9.22(18.60–54.00)	<0.001
	m/s	0.39 ± 0.24(0.03–1.49)	1.80 ± 0.64(0.09–2.77)	<0.001	0.30 ± 0.22(0.03–1.19)	1.07 ± 0.75(0.09–1.92)	<0.001	0.41 ± 0.21(0.06–1.20)	1.70 ± 0.56(0.12–2.48)	<0.001	0.49 ± 0.33(0.21–1.49)	2.08 ± 0.47(1.10–2.77)	<0.001
Eratio	kPa	2.28 ± 1.76(0.52–14.29)	7.44 ± 5.01(1.01–21.30)	<0.001	1.78 ± 0.73(0.62–4.08)	6.42 ± 6.35(1.01–19.30)	<0.001	2.36 ± 2.02(0.82–14.29)	8.10 ± 5.28(1.39–21.30)	<0.001	3.15 ± 2.12(0.52–8.34)	7.30 ± 4.56(1.47–17.45)	<0.001
	m/s	1.39 ± 0.43(0.46–3.70)	2.52 ± 0.93(1.00–4.56)	<0.001	1.27 ± 0.28(0.46–1.92)	2.23 ± 1.15(1.00–4.27)	<0.001	1.42 ± 0.46(0.86–3.70)	2.62 ± 0.91(1.17–4.56)	<0.001	1.57 ± 0.54(0.72–2.70)	2.55 ± 0.89(1.04–4.15)	<0.001

Emean = the mean elasticity; E_SD_ = standard deviation of the elasticity; Eratio = the ratio elasticity.

^*^Comparisons between benign and malignant lesions.

**Table 3:** Receiver operating characteristic curves for quantitative and qualitative parameters in the differentiation between benign and malignant lesions.

Variables		Cut-off value	Sensitivity (%)	Specificity (%)	Accuracy (%)	PPV (%)	NPV (%)	AUROC	95%CI	*P*
Emean	kPa	36.05	85.1 (40/47)	96.6 (172/178)	94.2 (212/225)	87.0 (40/46)	96.1 (172/179)	0.943	0.896–0.990	<0.001
	m/s	3.30	85.1 (40/47)	95.5 (170/178)	93.3 (210/225)	83.3 (40/48)	96.0 (170/177)	0.931	0.882–0.981	<0.001
E_SD_	kPa	17.90	93.6·(44/47)	97.8· (174/178)	96.9· (218/225)	91.7· (44/48)	98.3· (174/177)	0.936	0.866–1.000	<0.001
	m/s	0.91	93.6 (44/47)	95.5 (170/178)	95.1 (214/225)	84.6 (44/52)	98.3 (170/173)	0.936	0.866–1.000	<0.001
Eratio	kPa	3.67	76.6 (36/47)	91.0 (162/178)	88.0 (198/225)	69.2 (36/52)	93.6 (162/173)	0.872	0.809–0.936	<0.001
	m/s	1.87	76.6 (36/47)	91.0 (162/178)	88.0 (198/225)	69.2 (36/52)	93.6 (162/173)	0.863*	0.794–0.932	<0.001
Color pattern		2–3	89.4 (42/47)	97.2 (173/178)	95.6 (215/225)	89.4 (42/47)	97.2 (173/178)	0.957	0.914–1.000	<0.001

Emean = the mean elasticity; E_SD_ = standard deviation of the elasticity; Eratio = the ratio elasticity; PPV = positive predictive value; NPV = negative predictive value; CI = confidence interval; AUROC = area under the receiver operating characteristic curve.

^*^There are statistically significant difference between AUROC of color patterns and Eratio in speed mode (*P* = 0.03 < 0.05).

^·^E_SD_ expressed in KPa obtained the highest Sensitivity, Specificity, Accuracy, PPV, NPV compared with the Eratio with statistically significant difference. (Corresponding *P* = 0.008 for Sensitivity comparison, *P* = 0.002 for Specificity comparison, *P* = 0.000 for Accuracy comparison, *P* = 0.006 for PPV comparison, *P* = 0.030 for NPV comparison, all *P* < 0.05).

**Table 4:** Comparison of diagnostic performance among groups with different sizes.

Sizes	N	Variable	Cut-off (kPa)	Sensitivity (%)	Specificity (%)	Accuracy (%)	PPV (%)	NPV (%)	AUROC	95% CI	*P*
≤10mm	67	Emean	34.50	71.4 (5/7)	98.3 (59/60)	95.5 (64/67)	83.3 (5/6)	96.7 (59/61)	0.877	0–1.000	<0.001
10–20mm	108	Emean	36.05	87.5 (14/16)	97.8 (90/92)	96.3 (104/108)	87.5 (14/16)	97.8 (90/92)	0.930	0–1.000	<0.001
>20mm	50	Emean	52.05	83.3 (20/24)	96.2 (25/26)	90.0 (45/50)	95.2 (20/21)	86.2 (25/26)	0.962	0.902–1.000	<0.001

Emean = the mean elasticity; PPV = positive predictive value; NPV = negative predictive value; CI = confidence interval; AUROC = area under the receiver operating characteristic curve.
